# Dark Chocolate Intake Positively Modulates Redox Status and Markers of Muscular Damage in Elite Football Athletes: A Randomized Controlled Study

**DOI:** 10.1155/2018/4061901

**Published:** 2018-11-21

**Authors:** Elena Cavarretta, Mariangela Peruzzi, Riccardo Del Vescovo, Fabio Di Pilla, Giuliana Gobbi, Andrea Serdoz, Roberto Ferrara, Leonardo Schirone, Sebastiano Sciarretta, Cristina Nocella, Elena De Falco, Sonia Schiavon, Giuseppe Biondi-Zoccai, Giacomo Frati, Roberto Carnevale

**Affiliations:** ^1^Department of Medical-Surgical Sciences and Biotechnologies, Sapienza University of Rome, Latina, Italy; ^2^Villa Stuart Sport Clinics, FIFA Medical Center of Excellence, via Trionfale 5952, 00136 Rome, Italy; ^3^A.S. Roma Football Club, piazzale Dino Viola 1, 00128 Rome, Italy; ^4^Department of Medicine and Surgery, University of Parma, Ospedale Maggiore, Parma, Italy; ^5^Department of Physiology and Pharmacology, Sapienza University of Rome, Rome, Italy; ^6^IRCCS Neuromed, Pozzilli (IS), Italy

## Abstract

Intensive physical exercise may cause increase oxidative stress and muscular injury in elite football athletes. The aim of this study was to exploit the effect of cocoa polyphenols on oxidative stress and muscular injuries induced by intensive physical exercise in elite football players. Oxidant/antioxidant status and markers of muscle damage were evaluated in 24 elite football players and 15 controls. Furthermore, the 24 elite football players were randomly assigned to either a dark chocolate (>85% cocoa) intake (*n* = 12) or a control group (*n* = 12) for 30 days in a randomized controlled trial. Oxidative stress, antioxidant status, and muscle damage were assessed at baseline and after 30 days of chocolate intake. Compared to controls, elite football players showed lower antioxidant power and higher oxidative stress paralleled by an increase in muscle damage markers. After 30 days of dark chocolate intake, an increased antioxidant power was found in elite athletes assuming dark chocolate. Moreover, a significant reduction in muscle damage markers (CK and LDH, *p* < 0.001) was observed. In the control group, no changes were observed with the exception of an increase of sNox2-dp, H_2_O_2_, and myoglobin. A simple linear regression analysis showed that sNox2-dp was associated with a significant increase in muscle damage biomarker release (*p* = 0.001). An *in vitro* study also confirmed that polyphenol extracts significantly decreased oxidative stress in murine myoblast cell line C2C12-derived. These results indicate that polyphenol-rich nutrient supplementation by means of dark chocolate positively modulates redox status and reduced exercise-induced muscular injury biomarkers in elite football athletes. This trial is registered with NCT03288623.

## 1. Introduction

Intensive physical exercise may increase oxidative stress and cause muscular injury in elite athletes [1]. This represents a significant problem for professional football players representing more than one third of all time-loss injuries and causing more than a quarter of the total injury-dependent absence in high-level European professional football clubs [2]. Consequently, the overall burden of muscle injuries is the main reason for player unavailability in training and official matches for professional football clubs [3]. Accordingly, injuries during a regular season or a particular tournament can have considerable impact on team performance as well as on the economics of the club. Hereafter, prevention and reduction of muscle injuries in professional football should be of paramount importance.

The generation of reactive oxygen species (ROS) is a fundamental and physiological process of normal human biology. However, when ROS production and endogenous antioxidant ability are imbalanced, a maladaptive biological response occurs leading to both oxidative stress and inflammation [4, 5]. In muscle cells, aerobic energy production generates a significant amount of ROS, which can increase up to 10- to 20-fold during physical exercise [6]. Notably, previous evidence suggests that high ROS levels are able to induce muscular injury [1, 7, 8] with a consequent decrease in physical performance [9]. Several mechanisms contribute to the generation of ROS in skeletal muscles in response to intensive exercise. Among them, NADPH oxidase-derived formation of ROS may result in an altered redox state in muscles which may lead to contractile muscle dysfunction, accelerated muscle fatigue, longer recovery time, and reduced exercise performance [10].

Many studies have identified the potential antioxidant effect of polyphenols, a large group of natural compounds found in food and beverages [11]. Banerjee et al. highlighted that a supplementation with antioxidants is able to accelerate recovery from fatigue and to prevent exercise tissue damage [12]. Moreover, supplementation with other antioxidant nutrients, such as vitamin C and vitamin E, has been able to prevent exercise-induced oxidative damage but not inflammation in ultra-marathon runners [13]. Finally, previous work demonstrated a reduced exercise-induced muscular injury and a downregulation of monocytes expressing toll-like receptor 4 in kendo athletes by means of coenzyme Q10 administration [14, 15]. Overall, these data indicate that the administration of antioxidant nutrients may be a beneficial intervention to reduce the rate of muscular injury in endurance athletes. Cocoa may be appropriate for this purpose since it is a polyphenol-rich nutrient eliciting antioxidant effects [16]. A recent work demonstrated that acute cocoa-derived flavonoid intake may reduce oxidative stress and muscle damage induced by exercise [17]. However, there are few studies examining the effect of chronic cocoa flavonoid intake during exercise training.

Accordingly, the purpose of this study was to exploit the effect of chronic dark chocolate supplementation on muscular injury and oxidative stress during training exercise in elite football players.

## 2. Material and Methods

The study was performed on 24 young (17.2 ± 0.7 years) elite male football players during the first month of the regular season and 15 physically active male subjects who did not practice football but practice aerobic sports such as running, swimming, or gymnastics (engaging in at least 3-day·week^−1^ of moderate-to-intense physical activity, ranging from 3.0 to 6.0 METs and/or >6.0 METs) (24.8 ± 3.5 years) (Table 1). Players had at least 10 years of previous football training and were members of the Italian first-league A.S. Roma youth (17-19 years) team (Primavera). They were engaged in a 120-minute training (including a 15-minute warm-up, 30-minute technical/tactical skills, 30-minute aerobic training reaching 75% of the maximal heart rate, 30-minute strength training, and 15-minute cooldown) 6 times per week and a 90-minute match per week. The training program of the youth team was the same of the first A.S. Roma team (Serie A league). During the study, two players of the youth team also made their debut in the Serie A league with the first team and played regularly with the first team.

All participants and the head coach were explained the study's purposes, risks, and benefits; they were familiarized with the study's protocol during the pre-season screening; and they gave a written informed consent. The institutional review board approved this study (C.E. 4662), and the randomized controlled trial was registered on ClinicalTrials.gov (Identifier: NCT03288623).

Elite male athlete volunteers, aged between 18 and 35 years, were included in the study. Subjects were excluded if they suffer from an allergy to cocoa or any of the ingredients contained within either of the chocolate bars; they have a low platelet count (<170 × 10^09^/L); they are taking aspirin or aspirin-containing drugs, other anti-inflammatory drugs, or any drugs or herbal medicines known to alter platelet function or the haemostatic system in general (without a minimum washout period of one month); they are taking fish oils or evening primrose oil, or fat-soluble vitamin supplements within the last 4 weeks; they have unsuitable veins for blood sampling and/or cannulation; they have a BMI below 18 or above 35 kg/m^2^; they are taking any medicine known to affect lipid and/or glucose metabolism; they are suffering from alcohol or any other substance abuse or are having eating disorders; they have any known clinical signs of diabetes, hypertension, renal, hepatic, and haematological diseases, gastrointestinal disorders, endocrine disorders, coronary heart disease, infection, or cancer.

### 2.1. Study Design

In the first phase, we performed a cross-sectional study to compare oxidative stress, as assessed by blood levels of soluble Nox2 (sNox2-dp), H_2_O_2_ production, H_2_O_2_ breakdown activity (HBA) which is a method evaluating the antioxidant capacity of serum, and markers of muscle damage such as creatine kinase (CK), lactate dehydrogenase (LDH), and myoglobin in 24 young well-trained male elite football athletes and 15 sex-matched amateur controls. In the second phase, we performed a randomized controlled trial in elite football athletes to investigate the effect of daily supplementation with normal diet and 40 g of dark chocolate (20 g every 12 h) vs normal diet, for 30 days, on markers of oxidative stress and muscle damage.

Elite football athletes were randomly allocated to a treatment sequence with normal diet plus 40 g/day of commercially available dark chocolate in tablet (cocoa solids >85%, cocoa mass, fat-reduced cocoa, cocoa butter, sugar, and vanilla) or normal diet for 30 days. The content of total polyphenols in the dark chocolate employed in our study was 799 *μ*g GAE/ml.

Blood levels of CK, LDH, myoglobin, total polyphenols, and oxidative stress biomarkers were assessed at baseline and at 30 days after the last ingestion of chocolate. During the trial, participants were required to follow a diet adjusted according to their anthropometric and clinical characteristics and to the amount of calories coming from chocolate intake; furthermore, participants were restrained by having foods with high polyphenol content (blueberry, sweet cherry, strawberry, blackberry, red raspberry, chestnut, black tea, green tea, pure apple juice, hazelnut, red wine, and pomegranate juice) and/or additional chocolate.

Blood samples were collected in the morning (between 08 : 00 and 09 : 00 hours) after a fasting period of 8 h at baseline and 30 days after the last ingestion of chocolate.

### 2.2. Randomisation and Blinding

An individual not involved in the study assigned codes to the study treatments, randomly allocated the participants to a treatment sequence with normal diet plus dark chocolate or normal diet, and kept the key in a sealed envelope. The randomisation was carried out by a procedure based on a random numeric sequence. The authors and laboratory technicians were unaware of the treatment allocation.

### 2.3. Sample Size, Randomisation, and Blinding

Sample size calculation was computed with G^∗^Power [18] based on preliminary data, by means of a two tailed one-sample Student *t*-test with Welch correction: 1,69 (d) as effect size for sNox2-dp, 0.05 (a) as type I error probability, and 0.90 as power 1-b. Therefore, at least 22 elite soccer players (11 athletes per group, allocation ratio = 1) need to be randomly assigned to dark chocolate implementation or normal diet.

### 2.4. Extraction of Phenolic Fraction from Chocolate and Total Polyphenol Content Evaluation

One g of chocolate was weighted, and fat was removed by using 1 ml of n-hexane. Polyphenols were extracted from the defatted pellet using a total volume of 3 ml (1 × 3 ml) with 80% (*v*/*v*) of acetone : water at 80°C. This aqueous acetone solution, which contained most of all polyphenols, was used for polyphenols analysis and for *in vitro* study.

Total polyphenol content in extracted phenolic fraction from chocolate was evaluated by a modified Folin–Ciocalteu colorimetric method [19] and in plasma samples by the Folin–Ciocalteu method according to Serafini et al. [20]. Results were expressed as *μ*g gallic acid equivalent (GAE)/ml.

### 2.5. Extraction and Quantification of Catechin and Epicatechin from Chocolate

Catechin and epicatechin were extracted and quantified according to Gottumukkala et al. [21]. Briefly, about 10 g of chocolate sample was extracted with methanol on a hot water and all methanolic fractions were combined, filtered, and evaporated. Catechin and epicatechin stock solutions were prepared in methanol in the concentration range of 100–600 *μ*g/ml. HPLC analysis was performed using an HPLC system (Agilent 1200 Infinity Series HPLC system, Santa Clara, USA). The column temperature was maintained at 30°C. The HPLC mobile phase was as follows: solution A, 0.1 ml of orthophosphoric acid dissolved in 900 ml of HPLC-grade water; solution B, acetonitrile. The mobile phase was run using a gradient elution: at the time of 0.01 minutes, 11% B; at the time of 30 minutes, 25% B; at the time of 35 to 39 minutes, 100% B; and at the time of 40 to 50 minutes, 11% B. The mobile phase flow rate was 1.0 ml/minute, and the injection volume was 10 *μ*l. The eluents were detected and analyzed at 280 nm.

### 2.6. Plasma Extraction and Quantification of Epicatechin

Plasma samples were extracted by the method described by Spadafranca et al. [22]. Briefly, 20 *μ*l vitamin C-EDTA (200 mg vitamin C and 1 mg EDTA in 1 ml 0.4 mol/l NaH_2_PO_4_) and 20 *μ*l glucuronidase/sulfatase type II (Sigma, St. Louis, MO) were added to 200 *μ*l plasma and incubated at 37°C for 45 minutes. Flavonoids were extracted by addition of 500 *μ*l acetonitrile, and the mixture was centrifuged at 10,000 g for 5 minutes at room temperature. After centrifugation, the supernatant was removed, dried under nitrogen, and reconstituted in the aqueous HPLC mobile phase. After centrifugation, 50 *μ*l supernatant was injected into the HPLC column for separation, detection, and analysis.

The HPLC analysis was performed using an HPLC system (Agilent 1200 Infinity Series HPLC system, Santa Clara, USA). Separations were carried out at a flow rate of 1.5 ml/min with an isocratic mobile phase of 85% Na_2_PO_4_ 10 mol/l, pH 3, and 15% acetonitrile. Chromatograms were recorded at 279 nm, and plasma epicatechin identification was made by comparison of retention times with those of commercially available authentic (-)-epicatechin (Sigma, St. Louis, MO) through the same procedures as the plasma samples.

### 2.7. Determination of % HBA in Serum

Serum hydrogen peroxide (H_2_O_2_) breakdown activity (HBA) was measured with HBA assay kit (Aurogene, code HPSA-50). The % of HBA was calculated according to the following formula: % of HBA = [(Ac − As)/Ac] × 100 where Ac is the absorbance of H_2_O_2_ 1.4 mg/ml as is the absorbance in the presence of the serum sample.

### 2.8. Serum Nox2 Activation

Serum Nox2 was measured as soluble Nox2-derived peptide (sNox2dp) with an ELISA method, according to Pignatelli et al. [23].

### 2.9. H_2_O_2_ Production

Hydrogen peroxide (H_2_O_2_) was evaluated by a Colorimetric Detection Kit (Arbor Assays) and expressed as *μ*mol/l. Intra-assay and interassay coefficients of variation were 2.1% and 3.7%, respectively.

### 2.10. Muscle Damage Markers

Serum creatine kinase (CK), lactate dehydrogenase (LDH), and myoglobin levels were analyzed using a commercial ELISA kit (Antibodies, Germany; EIAab, China; DRG Instruments GmbH, Germany); the intra- and interassay coefficients were <10%.

### 2.11. In Vitro Study

#### 2.11.1. Cell Culture and Reagents

The murine myoblast cell line C2C12 was cultured in DMEM/20% heat-inactivated foetal bovine serum (FBS), 2 mM glutamine, and 1% antibiotics (all Gibco) for expansion and maintenance of the undifferentiating state. When cultures reached 80% confluence, myogenic differentiation was induced by replacing the expansion media with DMEM/0.2% FBS. Afterwards, cells were stimulated with H_2_O_2_ (5 mM, Solarbio, Beijing, China) alone or in combination with cocoa-derived polyphenols (50, 100, and 150 *μ*g/ml) for 30 minutes. Conditioned media were harvested and tested for the quantification of soluble Nox2 and H_2_O_2_ as described.

### 2.12. Statistical Methods

Continuous variables are reported as mean ± standard deviation unless otherwise indicated. Differences between categorical variables were tested using the *χ*^2^ test. The crossover study data were analyzed for the assessment of treatment and period effects, by performing a split-plot ANOVA with one between-subject factor (treatment sequence) and two within-subject factors (period 1 vs 2; pre- vs post-treatment). The full model was considered, allowing for the assessment of all main effects and interactions. Pairwise comparisons were corrected by *t*-test for paired data. Bivariate analysis was performed with the Spearman linear regression test. Multiple linear regression analysis was performed using a forward selection. *p* < 0.05 was considered statistically significant. All analyses were carried out with SPSS V.18.0 (Armonk, USA).

## 3. Results

### 3.1. Cross-Sectional Study

**Figure 1:**
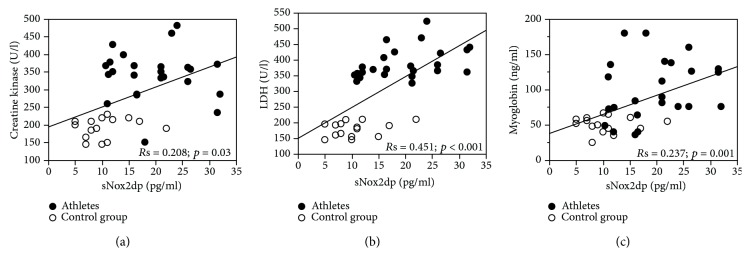
Linear correlation between sNox2-dp and creatine kinase (a), between sNox2-dp and LDH (b), and between sNox2-dp and myoglobin (c) in 15 controls (circle empty mark) and 24 elite football players (circle full mark).

Clinical characteristics of elite football players and controls are reported in Table 1. WBC, BMI, and HBA were higher in controls compared to elite football athletes. Conversely, LDH, CK, myoglobin sNox2-dp, and H_2_O_2_ were higher in elite athletics compared to controls (Table 1). A simple linear regression analysis showed that sNox2-dp was associated with CK (*R* = 0.208; *p* = 0.03), LDH (*R* = 0.451; *p* < 0.001), and myoglobin (*R* = 0.237; *p* = 0.001) (Figures 1(a)-1(c)).

### 3.2. Randomized Controlled Trial

Total polyphenol, catechin, and epicatechin contents of dark chocolate are reported in Table 2.

**Figure 2:**
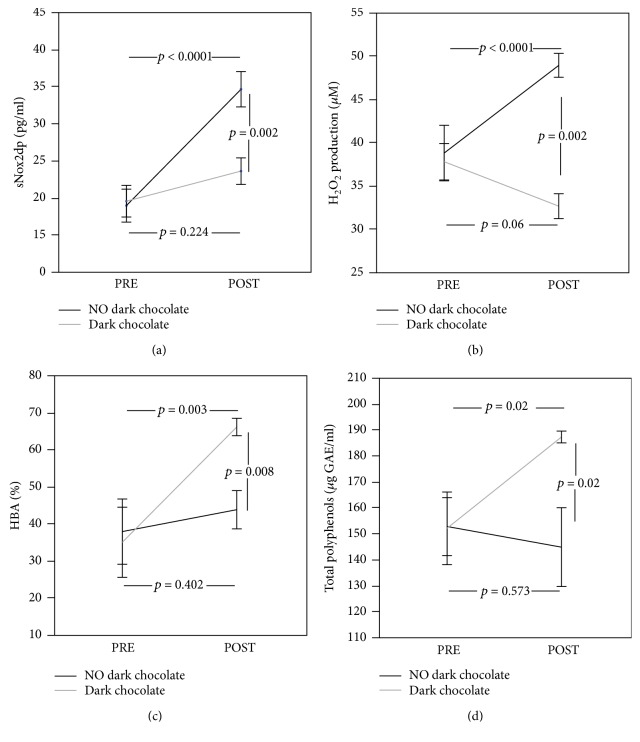
sNox2-dp levels (a), H_2_O_2_ production (b), hydrogen peroxide breakdown activity (HBA) (c), and total polyphenol (d) before and 30 days after daily intake of 40 g of dark chocolate (grey line) or without dark chocolate (black line) in elite football players.

**Figure 3:**
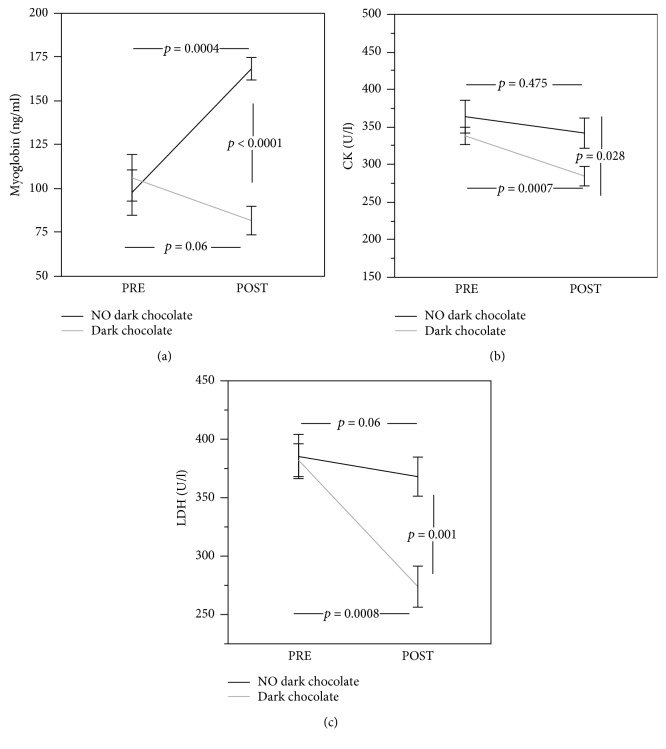
Myoglobin (a), creatine kinase (CK) (b), and lactate dehydrogenase (LDH) (c) concentration before and 30 days after daily intake of 40 g of dark chocolate (grey line) or without dark chocolate (black line) in elite football players.

No significant differences between clinical characteristics and biochemical parameters were found at baseline in the elite athlete groups allocated to dark chocolate intake and no dark chocolate intake (Table 3, Figures 2 and 3). Four athletes dropped out of the study to transfer to another team.

After 30 days of training, the control group showed increased levels of sNox2-dp and H_2_O_2_ compared to baseline (from 18.9 ± 7.0 pg/ml to 34.6 ± 7.5 pg/ml, *p* < 0.0001, and from 38.7 ± 10.2 *μ*M to 48.9 ± 4.3 *μ*M, *p* < 0.0001, respectively) (Figures 2(a) and 2(b)). Conversely, HBA and plasma total polyphenols resulted to be unchanged (from 37.78 ± 27.8% to 43.7 ± 16.3%, *p* = 0.402, and from 152.6 ± 35.3 *μ*g GAE/ml to 144.7 ± 48.06 *μ*g GAE/ml, *p* = 0.573, respectively) (Figures 2(c) and 2(d)). The pairwise comparisons showed that sNox2-dp levels and H_2_O_2_ production did not change after 30 days of dark chocolate intake compared to baseline (from 19.5 ± 6.7 pg/ml to 23.55 ± 5.6 pg/ml, *p* = 0.224, and from 37.7 ± 6.7 *μ*M to 32.6 ± 4.5 *μ*M, *p* = 0.06, respectively) (Figures 2(a) and 2(b)). Conversely, HBA and plasma total polyphenols increased after treatment (from 34.9 ± 29.8% to 66.0 ± 7.3%, *p* = 0.003, and from 151.9 ± 44.2 *μ*g GAE/ml to 187.1 ± 7.1 *μ*g GAE/ml, *p* = 0.02, respectively) (Figures 2(c) and 2(d)).

A significant difference between the two treatments (no dark chocolate vs dark chocolate) was found regarding sNox2-dp (34.6 ± 7.5 pg/ml vs 23.55 ± 5.6 pg/ml, *p* = 0.002) (Figure 2(a), H_2_O_2_ production (48.9 ± 4.3 *μ*M vs 32.6 ± 4.5 *μ*M, *p* < 0.002) (Figure 2(b), HBA (43.7 ± 16.3% vs 66.0 ± 7.3%, *p* = 0.008) (Figure 2(c)) and total polyphenols (144.7 ± 48.1 *μ*g GAE/ml vs 187.1 ± 7.16 *μ*g GAE/ml, *p* = 0.02) (Figure 2(d)).

Serum *muscle enzymes* highlighted in the control group increased myoglobin levels (from 97.4 ± 40.8 ng/ml to 168.0 ± 20.2 ng/ml, *p* = 0.0004), and there was no change in CK and LDH levels (from 363.0 ± 69.1 U/l to 341.1 ± 63.67 U/l, *p* = 0.475, and 384.7 ± 59.6 U/l to 367.5 ± 52.9 U/l, *p* = 0.06, respectively) (Figures 3(a)-3(c)). Conversely, we observed a significant decrease of CK (from 337.4 ± 36.5 U/l to 283.9 ± 41.0 U/l, *p* = 0.0007) and LDH (from 381.6 ± 44.3 U/l to 273.4 ± 55.5 U/l, *p* = 0.0008) (Figures 3(b) and 3(c)) after dark chocolate treatment. Myoglobin levels decreased without reaching statistical significance (from 105.8 ± 41.9 ng/ml to 81.4 ± 25.7 ng/ml, *p* = 0.06) (Figure 3(a)). A significant difference between the two treatments (no dark chocolate vs dark chocolate) was found regarding myoglobin (168.0 ± 20.3 ng/ml vs 81.4 ± 25.7 ng/ml, *p* < 0.001) (Figure 3(a)), CK (341.1 ± 63.67 U/l vs 283.9 ± 41.1 U/l, *p* = 0.028) (Figure 3(b)), and LDH (367.5 ± 53.0 U/l vs 273.4 ± 55.5 U/l, *p* = 0.001) (Figure 3(c)).

To assess the adherence to the protocol, we analyzed the levels of epicatechin which is a major component of dark chocolate. The results showed a significant increase of epicatechin in the dark chocolate group compared to the control group (189.8 ± 54.0 ng/ml vs <10 ng/ml, *p* < 0.0001).

A sensitivity analysis was then conducted by using generalized estimating equations (GLM), and point estimates of effect, 95% confidence intervals, and corresponding *p* values were reported. Chocolate exerted a significant beneficial effect on the following several variables at repeated measurement: sNox2-dp (point estimate of effect = −11.6 [95% -18.8; -4.4] pg/ml, *p* = 0.002), H_2_O_2_ (point estimate of effect = −15.2 [95% -23.3; -7.2] *μ*M, *p* < 0.0001), HBA (point estimate of effect = 25.2 [95% 3.8; 46.5] %, *p* = 0.021), total polyphenols (point estimate of effect = 43.1 [95% 7.6; 78.6] *μ*g GAE/ml, *p* = 0.01), CK (point estimate of effect = −31.6 [95% -52.8; -10.3] U/l, *p* = 0.004), LDH (point estimate of effect = −91.0 [95% -153.8; -28.1] U/L, *p* = 0.005), and myoglobin (point estimate of effect = −95.0 [95% -126.7; -63.2] U/L, *p* < 0.001).

No significant correlation was found between baseline blood parameters and days of unavailability days of FKT and occurrence of muscular and joint lesions (all *p* > 0.05), with the notable exception of baseline myoglobin levels, which were lower in subjects requiring more FKT afterwards (spearman rho = −0.553, *p* = 0.011).

No significant effect of chocolate intake was found on days of unavailability, days of FKT, and occurrence of muscular and joint lesions (all *p* > 0.05). Accordingly, changes in blood parameters were not significantly associated with days of unavailability, FKT, and occurrence of muscular and joint lesions (all *p* > 0.05).

### 3.3. In Vitro Study

**Figure 4:**
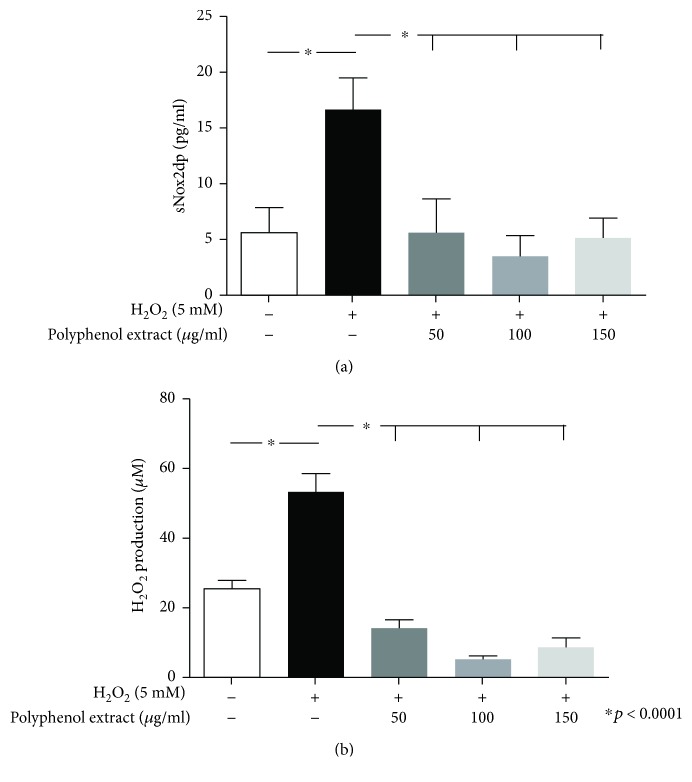
sNox2-dp levels (a) and H_2_O_2_ production (b) in murine myoblast cell line C2C12 stimulated with H_2_O_2_ (5 mM), alone or in combination with cocoa-derived polyphenols (50, 100, and 150 *μ*g/ml). All values are expressed as means ± SD.

In order to corroborate the clinical effects of cocoa-derived polyphenols on muscle redox state, we performed an in vitro study with a polyphenol extract at concentrations (50-150 *μ*g/ml) relatively similar to that found in serum of elite football athletes after dark chocolate intake. Murine myoblast cell line C2C12-derived conditioned media after stimulation with H_2_O_2_ showed both enhanced levels of Nox2 activation and H_2_O_2_ production (Figures 4(a) and 4(b)). Polyphenol extract significantly decreased Nox2 activation and H_2_O_2_ production (Figures 4(a) and 4(b)).

## 4. Discussion

This study showed that (1) oxidative stress and markers of muscle damage are significantly increased in elite football players compared to controls and (2) chronic intake of dark chocolate is able to reduce oxidative stress and muscle damage biomarkers during elite football players' training session.

The novel finding of the present study is the improvement of oxidative stress and muscle damage enzymes after 30 days by ingestion of dark chocolate in elite football athletes during intensive physical exercise. The effect of dark chocolate supports the hypothesis that polyphenol content, in particular epicatechin, may be responsible for this effect. Accordingly, total polyphenol content and epicatechin plasma levels were increased in the group of athletes randomized for dark chocolate intake.

The scientific background of our research was based on the evidence that intensive physical exercise implies a wide range of multifaceted biological activities challenging the physiological homeostasis of the body. The relationship between exercise and oxidative stress is extremely complex and mainly depends on mode, frequency, intensity, and duration of exercise. On the one hand, several experimental and epidemiological evidences have underlined the key role of physical exercise (PE) in decreasing oxidative stress, especially if associated to aging, and to prevent and positively modulate cardiovascular-associated risk factors [24, 25]. It was suggested that the reduction of oxidative stress triggered by PE could be associated with the improvement of the nitric oxide function. Regular exercise was also recently demonstrated to activate eNOS and nitrite production and to reduce oxidative stress in spontaneously hypertensive rats [26]. Moreover, PE was suggested to exert cardioprotective effects in ischemic rats in which high-intensity interval training increases NO metabolite levels and reduce myocardial infarction [27]. Oppositely, other reports have demonstrated that exercise increases the production of ROS, particularly suggesting that high-intensity, but not moderate, physical exercise can cause redox imbalance overwhelming the antioxidant defence ability, leading to several types of injuries [12, 28–30]. These emerging data have nonnegligible theoretical and applicative implications.

According to this premise, professional training programs including those for elite football players could indirectly and physiologically induce oxidative stress in athletes and significantly influence biological antioxidant patterns. Accordingly, in our cross-sectional study, we found an increase in Nox2 activation and H_2_O_2_ production with reduced antioxidant property as indicated by decreased HBA in elite football players compared to controls. Moreover, at the same time, we observed an increase in muscle damage markers such as CK, LDH, and myoglobin.

These aspects represent a serious issue since a recent study showed that levels of oxidative stress markers are directly correlated with markers of muscular damage in elite football players playing in the Italian Serie A league [1].

For this reason, dietary regimens including antioxidant supplementation are now considered important interventions able to counteract the hazardous effects of free radicals by increasing the antioxidant profile and regulating the equilibrium between oxidant and antioxidant species [31]. These beneficial effects were observed in both animals and healthy subjects and in other chronic conditions that are accompanied by enhanced levels of oxidative stress [31–33]. Supporting endogenous defences with oral antioxidant supplementation may represent a suitable noninvasive tool for preventing or reducing oxidative stress during intensive physical training. The protective effects against oxidative stress elicited by antioxidant-rich nutrients have been partially ascribable to the high content of polyphenols (a class of chemicals characterized by the presence of phenol units in their chemical structure). The best-characterized biological property of all flavonoids as for polyphenols in general is their ability to act as antioxidants, by inhibiting ROS accumulation by scavenging ROS or by inhibiting enzymes involved in ROS production or by enhancing the natural antioxidant defences [34, 35]. In particular, a recent study suggests that daily supplementation with flavonol-rich cocoa over 4 weeks may improve oxidative stress biomarkers as indicated by an increase in total GSH levels and a decrease in urinary F2-isoprostane excretion [30].

We planned to use this body of evidence as a benchmark for the development of new strategies in the setting of elite athlete training programs. In this interventional study, we thus demonstrated for the first time that polyphenol-rich nutrient supplementation of dark chocolate reduces exercise-induced oxidative stress and muscular injury biomarkers in elite football athletes.

The antioxidative effects induced by cocoa-derived polyphenols were also confirmed on skeletal muscle cells *in vitro*. Our experiments were conducted using concentrations of polyphenol extract relatively close to that achieved in blood circulation of athletes after dark chocolate intake. Notably, the lower dose of polyphenols was able to completely counter the effects of induced oxidative stress, restoring the physiological redox state in treated murine myoblasts.

In conclusion, the present study provides the first direct relationship between cocoa-based polyphenol-rich nutrient supplementation and the effect of high-intensity training on elite athletes' antioxidant status. Based on our results, the development and improvement of training techniques focusing also on new nutrition strategies may help to reduce muscular damage in elite football players.

## Figures and Tables

**Table 1:** Baseline characteristics of controls and elite football players.

	Controls (*n* = 15)	Football elite players (*n* = 24)	*p*
Age (years)	24.8 ± 3.5	17.2 ± 0.7	<0.001
Gender (male/female)	15/0	24/0	1
WBC (×10^3^ *μ*l)	7.1 ± 1.4	5.6 ± 1.3	0.001
PLT (×10^3^ *μ*l)	233.8 ± 48.4	228 ± 39.7	0.357
RBC (×10^6^ *μ*l)	5.3 ± 0.3	5.8 ± 0.3	0.559
Cholesterol (mg/dl)	185.1 ± 30.8	172.3 ± 29.4	0.130
BMI	24.3 ± 1.4	22.5 ± 1.5	<0.01
Glycaemia (mg/dl)	89.0 ± 28.8	83.5 ± 15.2	0.276
LDH (U/l)	179.5 ± 23.5	387.0 ± 50.7	<0.01
CK (U/l)	192.3 ± 28.7	342.6 ± 70.2	<0.01
Myoglobin (ng/ml)	50.6 ± 11.3	100.1 ± 42.9	<0.01
sNox2-dp (pg/ml)	13.8 ± 7.7	19.5 ± 6.9	<0.01
H_2_O_2_ (*μ*M)	22.6 ± 13.2	38.8 ± 7.3	<0.0001
HBA (%)	52.9 ± 23.0	37.5 ± 11.4	<0.0001
Training per week (h)	7.2 ± 1.5	18 ± 2	<0.0001
Football practice (yrs.)	0	10 ± 1.2	<0.0001

WBC: white blood cells; PLT: platelet; RBC: red blood cells; BMI: body mass index; LDH: lactate dehydrogenase; CK: creatine kinase; HBA: hydrogen peroxide (H_2_O_2_) breakdown activity.

**Table 2:** Total polyphenol content in dark chocolate.

Compounds	Dark chocolate
Total polyphenols (*μ*g GAE/ml)	799
Epicatechin (mg/g)	0.65
Catechin (mg/g)	0.26

**Table 3:** Baseline characteristics of elite football players before treatment.

	No dark chocolate (*n* = 10)	Dark chocolate (*n* = 10)	*p*
Age (years)	17.0 ± 0.9	17.4 ± 0.5	0.859
Gender (male/female)	10/0	10/0	1
WBC (×10^3^ ml)	5.0 ± 1.3	6.2 ± 1.1	0.983
PLT (×10^3^ ml)	226.6 ± 55.4	230.0 ± 12.8	0.580
RBC (×10^6^ ml)	5.3 ± 0.3	5.5 ± 0.3	0.950
LDL (mg/dl)	82.9 ± 18.3	84.3 ± 22.2	0.566
Glycaemia (mg/dl)	84.0 ± 11.2	83.1 ± 19.1	0.448
LDH (U/l)	389.7 ± 59.9	384.2 ± 42.2	0.398
CK (U/l)	363 ± 21.83	341.1 ± 20.13	0.880
Myoglobin (ng/ml)	97.4 ± 40.8	105.8 ± 41.9	0.579
hs-PCR (mg/l)	0.6 ± 0.4	0.7 ± 0.6	0.759
sNox2-dp (pg/ml)	18.9 ± 7.0	19.5 ± 6.7	0.844
H_2_O_2_ (*μ*M)	38.7 ± 10.1	37.7 ± 6.6	0.746
HBA (%)	37.8 ± 27.8	34.9 ± 29.8	0.775

WBC: white blood cells; PLT: platelet; RBC: red blood cells; LDL: low-density lipoprotein; LDH: lactate dehydrogenase; CK: creatine kinase; HBA: hydrogen peroxide (H_2_O_2_) breakdown activity.

## Data Availability

The data used to support the findings of this study are available from the corresponding author upon request.

## References

[1] Becatti M., Mannucci A., Barygina V. (2017). Redox status alterations during the competitive season in élite soccer players: focus on peripheral leukocyte-derived ROS. *Internal and Emergency Medicine*.

[2] Ekstrand J., Hägglund M., Waldén M. (2011). Epidemiology of muscle injuries in professional football (soccer). *The American Journal of Sports Medicine*.

[3] Hägglund M., Waldén M., Magnusson H., Kristenson K., Bengtsson H., Ekstrand J. (2013). Injuries affect team performance negatively in professional football: an 11-year follow-up of the UEFA Champions League injury study. *British Journal of Sports Medicine*.

[4] Phaniendra A., Jestadi D. B., Periyasamy L. (2015). Free radicals: properties, sources, targets, and their implication in various diseases. *Indian Journal of Clinical Biochemistry*.

[5] Sies H. (2015). Oxidative stress: a concept in redox biology and medicine. *Redox Biology*.

[6] Sjödin B., Westing Y. H., Apple F. S. (1990). Biochemical mechanisms for oxygen free radical formation during exercise. *Sports Medicine*.

[7] Finaud J., Lac G., Filaire E. (2006). Oxidative stress: relationship with exercise and training. *Sports Medicine*.

[8] Peake J., Nosaka K., Suzuki K. (2005). Characterization of inflammatory responses to eccentric exercise in humans. *Exercise Immunology Review*.

[9] Radák Z., Pucsok J., Mecseki S., Csont T., Ferdinandy P. (1999). Muscle soreness-induced reduction in force generation is accompanied by increased nitric oxide content and DNA damage in human skeletal muscle. *Free Radical Biology & Medicine*.

[10] Powers S. K., Talbert E. E., Adhihetty P. J. (2011). Reactive oxygen and nitrogen species as intracellular signals in skeletal muscle. *The Journal of Physiology*.

[11] Manach C., Scalbert A., Morand C., Rémésy C., Jiménez L. (2004). Polyphenols: food sources and bioavailability. *The American Journal of Clinical Nutrition*.

[12] Banerjee A. K., Mandal A., Chanda D., Chakraborti S. (2003). Oxidant, antioxidant and physical exercise. *Molecular and Cellular Biochemistry*.

[13] Mastaloudis A., Morrow J. D., Hopkins D. W., Devaraj S., Traber M. G. (2004). Antioxidant supplementation prevents exercise-induced lipid peroxidation, but not inflammation, in ultramarathon runners. *Free Radical Biology & Medicine*.

[14] Kon M., Tanabe K., Akimoto T. (2008). Reducing exercise-induced muscular injury in kendo athletes with supplementation of coenzyme Q10. *The British Journal of Nutrition*.

[15] Shimizu K., Kon M., Tanimura Y. (2015). Coenzyme Q10 supplementation downregulates the increase of monocytes expressing toll-like receptor 4 in response to 6-day intensive training in kendo athletes. *Applied Physiology, Nutrition, and Metabolism*.

[16] Andújar I., Recio M. C., Giner R. M., Ríos J. L. (2012). Cocoa polyphenols and their potential benefits for human health. *Oxidative Medicine and Cellular Longevity*.

[17] González-Garrido J. A., García-Sánchez J. R., Garrido-Llanos S., Olivares-Corichi I. M. (2017). An association of cocoa consumption with improved physical fitness and decreased muscle damage and oxidative stress in athletes. *The Journal of Sports Medicine and Physical Fitness*.

[18] Faul F., Erdfelder E., Buchner A., Lang A. G. (2009). Statistical power analyses using G^∗^Power 3.1: tests for correlation and regression analyses. *Behavior Research Methods*.

[19] Mosca L., de Marco C., Visioli F., Cannella C. (2000). Enzymatic assay for the determination of olive oil polyphenol content: assay conditions and validation of the method. *Journal of Agricultural and Food Chemistry*.

[20] Serafini M., Maiani G., Ferro-Luzzi A. (1998). Alcohol-free red wine enhances plasma antioxidant capacity in humans. *The Journal of Nutrition*.

[21] Gottumukkala R. V. S. S., Nadimpalli N., Sukala K., Subbaraju G. V. (2014). Determination of catechin and epicatechin content in chocolates by high-performance liquid chromatography. *International Scholarly Research Notices*.

[22] Spadafranca A., Martinez Conesa C., Sirini S., Testolin G. (2010). Effect of dark chocolate on plasma epicatechin levels, DNA resistance to oxidative stress and total antioxidant activity in healthy subjects. *The British Journal of Nutrition*.

[23] Pignatelli P., Carnevale R., Cangemi R. (2010). Atorvastatin inhibits gp91phox circulating levels in patients with hypercholesterolemia. *Arteriosclerosis, Thrombosis, and Vascular Biology*.

[24] Conti V., Corbi G., Russomanno G. (2012). Oxidative stress effects on endothelial cells treated with different athletes’ sera. *Medicine & Science in Sports & Exercise*.

[25] Conti V., Russomanno G., Corbi G. (2013). Aerobic training workload affects human endothelial cells redox homeostasis. *Medicine & Science in Sports & Exercise*.

[26] Kumral Z. N., Sener G., Ozgur S. (2016). Regular exercise alleviates renovascular hypertension-induced cardiac/endothelial dysfunction and oxidative injury in rats. *Journal of Physiology and Pharmacology*.

[27] Fallahi A., Gaeini A., Shekarfroush S., Khoshbaten A. (2015). Cardioprotective effect of high intensity interval training and nitric oxide metabolites (NO2−, NO3 −). *Iranian Journal of Public Health*.

[28] Packer L., Cadenas E., Davies K. J. A. (2008). Free radicals and exercise: an introduction. *Free Radical Biology & Medicine*.

[29] Fisher-Wellman K., Bloomer R. J. (2009). Acute exercise and oxidative stress: a 30 year history. *Dynamic Medicine*.

[30] Metin G., Gümüştaş M. K., Uslu E., Belce A., Kayserilioglu A. (2003). Effect of regular training on plasma thiols, malondialdehyde and carnitine concentrations in young soccer players. *The Chinese Journal of Physiology*.

[31] Corbi G., Conti V., Komici K. (2018). Phenolic plant extracts induce Sirt1 activity and increase antioxidant levels in the rabbit’s heart and liver. *Oxidative Medicine and Cellular Longevity*.

[32] Davinelli S., Corbi G., Zarrelli A. (2018). Short-term supplementation with flavanol-rich cocoa improves lipid profile, antioxidant status and positively influences the AA/EPA ratio in healthy subjects. *The Journal of Nutritional Biochemistry*.

[33] Davinelli S., Corbi G., Righetti S. (2018). Cardioprotection by cocoa polyphenols and *ω*-3 fatty acids: a disease-prevention perspective on aging-associated cardiovascular risk. *Journal of Medicinal Food*.

[34] Forte M., Conti V., Damato A. (2016). Targeting nitric oxide with natural derived compounds as a therapeutic strategy in vascular diseases. *Oxidative Medicine and Cellular Longevity*.

[35] Loffredo L., del Ben M., Perri L. (2016). Effects of dark chocolate on NOX-2-generated oxidative stress in patients with non-alcoholic steatohepatitis. *Alimentary Pharmacology and Therapeutics*.

